# Explanatory hypotheses of the ecology of new clinical presentations of Dissociative Identity Disorders in youth

**DOI:** 10.3389/fpsyt.2022.965593

**Published:** 2022-10-10

**Authors:** Christophe Gauld, Pauline Espi, Olivier Revol, Pierre Fourneret

**Affiliations:** ^1^Service de Psychopathologie de l'enfant et de l'adolescent, Hôpital Femme Mère Enfant, Hospices Civils de Lyon, Lyon, France; ^2^UMR CNRS 8590 IHPST, Université de la Sorbonne, Paris, France; ^3^Laboratory of Social Neuroscience and Comparative Development, Institut des Sciences Cognitives, UMR5229, Université Lyon 1, Lyon, France

**Keywords:** Dissociative Identity Disorder, dissociation, adolescent, nosology, classifications, self-diagnosis, iatrogenicity

## Abstract

Dissociative Identity Disorders (DIDs) are controversial psychiatric conditions encountered in clinical practice and nosology. DID as described in the international classifications has little similarity with the clinical picture of “DID” met in current youth psychiatry. From this Perspective, we hypothesize that this current clinical presentation does not satisfy the categorical criteria of the international classifications. Based on the two terminological challenges related to the definition of DID (i.e., the notion of *dissociative disorders* and the different meanings of the term *identity*), we propose to differentiate two distinct entities from each other. The first is medical and listed in diagnostic criteria of international classifications; the second comes from popular culture and refers to the vast majority of clinical presentations received in daily clinical practice—presented under the term Dissociative Identity Conditions (DIC). Since the status of DIC is a hot topic in current clinical psychiatry, we aim to identify eight possible explanations that can be provided to support its occurrence: (1) impact of iatrogenicity; (2) factors of suggestibility and desire for social acceptability; (3) psychoanalytic explanations; (4) neuropsychological explanations; (5) socio-cognitive explanations; (6) emotional labeling; (7) narrative explanations; (8) and transient illnesses explanations. In conclusion, we sustain that DIC results from a narrative interpretation of medical discourse by popular culture, developing in patients presenting undeniable distress. Such a transient disease fits in an ecological niche, which echoes the values of society, persisting under the action of a need for narrative continuity of the self.

## Introduction

Dissociative Identity Disorder (DID) is included in the International Classification of Diseases, Eleventh Edition (ICD-11) in the sub-chapters of dissociative disorders (1). As demonstrated by a large body of literature that dates back more than 50 years (2–4), the definition of DID within the ICD-11 raises a certain number of difficulties since it is based on a large number of unexplained notions, e.g., identity, personality state, sense of self, agency, phenomenological experience, self-control, consciousness, social interactions, alterations of various neurocognitive modules, and amnesia (1).

Relatively similarly, the Diagnostic and Statistical Manual of mental disorders, fifth edition (DSM-5) describes DID as an identity disturbance characterized by distinct personality states and discontinuity in sense of self and agency, with an alteration of modules of thought, behavior, perception of memory, and associated with a criterion of clinical significance witnessing distress or impaired functioning, in the absence of substance use or other cultural or religious practices (5). DID is described in the DSM-5 as regularly related to traumatic episodes, and strongly dependent on the two expressions of dissociation which refers both to depersonalization (experience of reality or detachment from one's mind, oneself, or body) and derealization (experience of reality or detachment from the outside world).

A practical challenge adds to the complexity of these definitions as the DID described in the international classifications has little similarity with the clinical picture of “DID” encountered in current adolescent psychiatry clinical practice (6), which has appeared in young self-diagnosed patients. The relatively recent increase in clinical consultations for “DID” raises several observations: whether or not this clinical presentation corresponds to a diagnostic category as described in the international nosographies (1, 5); the influence of the media or of the healthcare environment (including iatrogenicity) (7); the importance that “DID” appears in suggestible people, eager for social acceptability (8), or in individuals with a tendency to fantasize (9).

Based on these observations, the hypothesis of this Perspective is based on the clinical observation that the current clinical presentation of “DID” does not meet the categorical criteria of the international classifications, despite them presenting themselves as “patients with a DID.” Although potentially experiencing episodes of dissociation, these “DID” refer to self-diagnosed patients claiming to be experts in their own suffering and asking for medical recognition. These clinical presentations encountered in the youth clinical practice first and specifically lead to diagnoses of “DID” without any medical evaluation having been carried out (referring to a self-diagnosis). In this way, the debate on these conditions goes further than the (more or less) bad fit of these current clinical pictures with international criteriology: it concerns the deeper problem of self-diagnosed conditions, with strong narrative components and labeling of emotions, related to self-categorizations transforming the way people perceive themselves. Like the DID described in the DSM-5, these are located in a (“ecological”) historical and temporal context; one of the main questions that this article seeks to answer is why adolescents land on DID as part of a desire for socialization and integration into their social community. These actual cases show a strong desire to socialize and integrate into their community, pushing them to build a complex discourse around the theme of DID. It is interesting to see that the rejection of professionals, who refuse to listen to them, constitutes one of the red flags, as described below. Also, these patients with DID present themselves to clinicians and their entourage without shame, and without assessable amnesia, describing their condition based on clinical knowledge learned on networks and social media. Unlike DID, they raise questions other than the relationship to memory, iatrogenic, criteriology, or diagnostic reliability, etc.—as we will describe later.

With these clinical pictures, there is no confusion with the (Schneiderian) experiences of psychosis. The DSM-5 suggests that DID may have psychotic disorders as differential diagnoses, including Schneiderian first-rank symptoms (e.g., auditory hallucinations, thought broadcast, thought insertion, thought withdrawal, or delusional perception). However, the patients described here have no such symptoms, and no more loss of sense of agency, nor ego-dystonic and puzzling emotions. Moreover, the absence of these psychotic-like symptoms allows us to better affirm the specificity of these conditions which should be considered in all their originality. However, while psychotic symptoms are not present, the disturbance of the sense of self, related to a potential dissociation, may be minimally present. This presence confirms the relation of these conditions with the notion of dissociative identity. We would like to clarify that we are discussing a clinical practice of adolescent psychiatry concerning DID, which could not be applied to the clinical practice of adult psychiatry. More generally, the scope of the opinions and arguments of this paper should therefore not be transposed to adult psychiatric clinical practice.

Finally, the objective of this article is not to complicate the debate concerning the difficulties raised by the DID for more than 100 years (10). Rather, its purpose is to first identify the challenges related to the clinical presentation of “DID,” and then discuss the potential explanatory hypotheses for the emergence of these self-diagnosed “DID.” In parallel, in order to bear witness to these emerging clinical pictures, we propose to isolate four elements: a clinical vignette, a list of the typical symptoms found in these patients, an example of their specific vocabulary, and a set of explanations for their definition of “DID” and their expression of symptoms. The following clinical picture (Table 1) helps to better understand the description of this emerging condition in contemporary psychiatry.

**Table 1:** Clinical vignette encountered in the current clinical practice of adolescent psychiatry.

In the current clinical practice of adolescent psychiatry, for a relatively short time in the history of Dissociative Identity Disorders, we encounter clinical pictures which do not correspond to any (criteriological but also phenomenological) description of the DSM-5, and which are not discussed in the psychiatric literature elsewhere. These clinical pictures refer to patients self-diagnosed, without any amnesia, and with weak dissociations. They know the DSM-5 criteria by heart, and they can recite them. We can see, however, that they have not read the detail of the DSM-5 which follows the list of criteria, i.e., the “Diagnostic Features,” the “Associated Features,” and the “Development and Course.” Many of them belong to a social media community discussing “DID,” guided by a limited number of well-identified mental health influencers. They do not present frank simulation or personality disorders (and in particular histrionic personality disorder). Moreover, in addition to the identity claim specific to adolescence, we find a set of symptoms belonging to clinical psychiatry, which does not correspond to any category, e.g., weak dissociations, anxiety, and feeling of unease, explicit harm (without a depressive or an anxiety disorder being diagnosed). As argued in this article, these clinical pictures fall within the scope of psychiatry but cannot be considered as disorders (but rather as conditions), representing a way of expressing a type of suffering in young people.

## Challenges with the definitions of dissociative identity conditions

Self-diagnosed patients currently seen in clinical practice report having read and learned the DSM criteria. This reading and this communication within the community of “DID” is problematic, in particular because of the equivocal definition of this disorder in the DSM. Two terminological challenges arise from the definition of DID, as described in the international classifications. First, the term “dissociative disorder” can be interpreted according to two hypotheses, leading to confusion in the definition. According to the first hypothesis, DID is a “dissociation-related disorder,” related to identity. In this sense, the problem is above all related to dissociation; identity is only affected secondarily since dissociation causes the loss of sense of identity. This hypothesis corresponds to the definition of DID that seems to be implied by the international classifications. According to the second hypothesis, DID is a disorder that “dissociates the identity” into several identities. This refers to the clinical presentations found in adolescent psychiatry, and themselves referring to their own lay understanding of DID (11).

The second terminological challenge corresponds to the two consensual definitions of identity that exist. First, identity can be understood as a similarity in terms of several characteristics. In this way, identity is defined as a set of characteristics that allows individualization from several elements. In DID, these elements include several behavioral and psychological functions (e.g., of the brain) that no longer work together, as described in the DSM-5: “[DID is] characterized by an unintended disruption or discontinuity in the normal integration of one or more of the following elements: identity, sensations, perceptions, affects, thoughts.” It is through this notion of identity that DID is medically understood. DID would then be a loss of the function of integration between different functions. However, secondly, identity can also be understood as what remains identical to oneself. This definition of identity refers to personal, cultural, or community identities. In this sense, identity characterizes individuality, an exclusivity for oneself. This identity is defined as a sense of stable belonging to oneself and/or to the community. We propose that it is through this notion of identity that DID is currently understood in contemporary popular culture. It is through this notion of identity that patients with a “DID” currently present themselves in clinical practice.

In summary of this first section, we propose two definitions of DID:

- The first is medical and listed in the diagnostic criteria of international classifications: it is based on the first definition of identity and refers to a DID that considers identity as an inability to be one with several elements. The dissociation that characterizes this type of DID is related to functions being independent of each other.
- The second definition comes from popular culture: it is based on the second definition of identity and corresponds to the presentations encountered in the clinical practice of adolescent psychiatry. It refers to a “DID” that considers identity as the inability to belong to oneself or to the community. “DID” corresponds to the vast majority of clinical presentations received in daily clinical practice. Table 2 illustrates the community lexicon that clinicians might need when encountering individuals with “DID.”

**Table 2:** Community lexicon.

	**Name**	**Definition**
Lexicon	Alter (formerly: Alter-personality)	One of the identities (12)
	Host system	Set of alters
	Front	Alter visible (temporary status)
	Co-front	Front of several alters
	Switch	Change of alters
	Blurring (or switchy-switchy)	Rapid changes between alters that can go as far as overlapping and the absence of demarcation between alters
	Singlet	Non-multiple person
	Innerworld	Underlying space where alters “go” when they are not psychically front and center (seems “visitable” on request)
Alter roles	Protector	Alter who provides protection for oneself or others
	Host	Alter who most commonly fronts
	Social	Alter who interacts with the environment
	Persecutor	Protector “in their own way”
	Caregiver	Alter who takes care of the body
	Trauma holder	Alter who keeps traumas
Alter styles	Little	Alter is a young alter
	Factive	Alter is a a person
	Fictional	Alter is a fictional character
	Non-human	Could be an animal or a mythological character, etc.

Since this emerging patient population is self-diagnosed, and because they belong to an adolescent community, a specific and remarkable vocabulary should be recognized by the clinician. This vocabulary can serve as red flags to identify the patients most at risk of presenting with “DIC”.

Note however that the main goal of this opinion article is not to systematically search for the lack of fit between these clinical pictures and the DSM-5 criteria. Rather, the enlightenment of a mismatch with the classification is only a tool to highlight that these clinical pictures do not correspond to the description of DID in the scientific psychiatric literature (that it deals with the phenomenological description or with the criteriology of DID).

**Figure 1:**
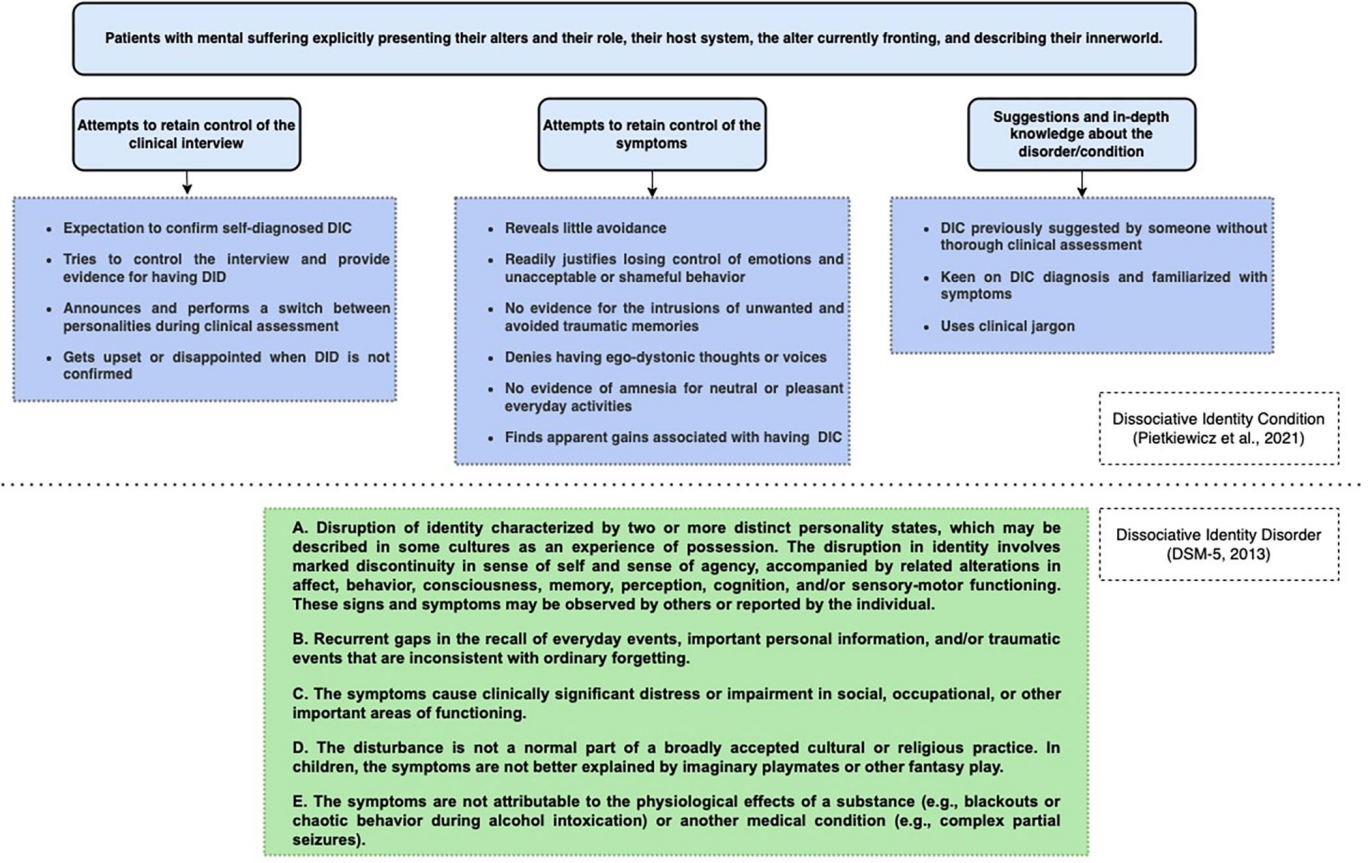
Details of the three categories allow clinicians to differentiate between the DID describe in the international classifications and the “DID” encountered in clinical practice. Restructured from (6). See Table 2 for the details of the terms used.

We argue that these two definitions lead us to consider two entities that should themselves be distinguished from each other. At least five questions arise from this distinction. The first corresponds to the mismatch between the clinical phenotype of patients and the criteria of international classifications. Clinically, these patients are in the quest for an identity, an essential issue of adolescence seeking to integrate the cognitive, instrumental, affective, and relational dimensions (13). The exploration of different identities could therefore constitute a constructive step, which requires openness to the acceptance of the diversity of identities, in particular through peer recognition. The second question concerns the existence of a dissociation in “DID.” Undeniably, a number of young patients with “DID” seen in clinical practice have a history of trauma (14, 15); a dissociation could therefore be present. We assume that the intensity of the dissociation is a continuum, with some patients experiencing major dissociative episodes and others minor ones. The most important dissociations are related to greater harm and more mental suffering (16). Our hypothesis is that the dissociation experienced in the clinical form of “DID” would be less important than the dissociation experienced in DID (17, 18). The third question corresponds to the clinician's ability to differentiate “DID” currently encountered in clinical practice from DID described in international classifications. A significant body of literature, and in particular red flags, have been proposed to differentiate between these two types of entities (6). Pietkiewicz et al. (6) propose five salient themes identified in these patients during an interpretative phenomenological analysis: endorsement and identification with the diagnosis, using the notion of dissociative parts to justify identity confusion and conflicting ego-states, gaining knowledge about the condition affects the clinical presentation, fragmented personality becomes an important discussion topic with others, and ruling out the condition leads to disappointment or anger. For the sake of clarity, we propose to structure these phenomenological data, corresponding to these red flags (6), into three main categories, allowing us to differentiate these two presentations of DID: (i) attempts to retain control of the clinical interview, (ii) attempts to retain control of the symptoms, (iii) and suggestions and in-depth knowledge about the entities (Figure 1).

The fourth question seeks to identify why this emerging type of dissociative disorder is centered around *identity*, and not, for instance, around dissociative fugue. With regard to dissociative fugue, an “epidemic” of this psychiatric disorder had been described around 1900, especially when tourism and vagrancy were socially and culturally valued (10). In parallel, today, the question of identity is socially highlighted. Thus, we identify at least four contemporary factors relating to an injunction to be oneself: a cult of personal development (19), praise of introspection, an indisputable value of openness to diversity, and individualistic empowerment. These different factors could explain why this dissociative disorder takes a form centered around identity and not around other social values (like fugue or travel). Finally, the fifth question seeks to ascertain whether this clinical “DID” is a disorder or only a *condition*, for which any risk of over-medicalization and over-treatment should be avoided. This question refers to a debate seeking to differentiate the normal from the pathological, in particular anchored around normativism (20) and naturalism (21). One of the most consensual answers to this debate for psychiatry is related to psychiatric disorders being the phenotypic expression of dysfunctions that directly lead to harm, e.g., distress or a disability (22). In the absence of dysfunction and harm, in the Discussion section of this Perspective, we will refer to “DID” as a Dissociative Identity Condition (DIC).

In order to guide the clinician to deal with these relatively new clinical pictures, we propose to open up some avenues of explanation to better understand these self-diagnosed clinical presentations.

## Discussion: Challenges related to the potential explanatory hypotheses of the dissociative identity conditions

The status of DIC remains a hot topic in clinical psychiatry (3, 11, 23–25). We identified eight possible explanations that can be provided to support the occurrence of DIC, which in a pluralistic framework should all be considered: (1) impact of iatrogenicity; (2) factors of suggestibility and desire for social acceptability; (3) psychoanalytic explanations; (4) neuropsychological explanations; (5) socio-cognitive explanations; (6) emotional labeling; (7) narrative explanations; (8) and transient illnesses explanations.

### Impact of iatrogenicity

A number of empirical data suggest that patients with DIC have a more explicit clinical presentation after medical interviews. Several hypotheses can be made to explain this phenomenon, such as increased awareness of the disorder, a fascination for DIC by health professionals (26), an influence of dissociative therapeutics (e.g., hypnosis), or the exaggerated establishment of causal relationships between childhood maltreatment and dissociative symptoms (27, 28).

### Suggestibility factors and desire for social acceptability

It could be argued that patients with DIC would have a tendency toward exaggeration or excessive imagination, an excess of suggestibility, or cognitive distortions (29, 30). However, empirical research does not show a relationship between dissociation and suggestibility, nor a relationship between phantasmagoria (or imagination) and psychopathology (31). For example, patients with a supposed DID (according to the Structured Clinical Interview for DSM-IV Dissociative Disorders-Revised—SCID-D) and actors mimicking the criteria of DID show significant differences in functional Magnetic Resonance Imaging and Positron Emission Tomography (32, 33).

### Psychoanalytic explanations

A psychoanalytic explanation sustains that the DIC would be only the symptomatic, defensive, and rationalizing expression of a form of desirability and the will for integration and social validation, passing through detachment from the body (34). The adolescent population has its own policy, in particular through the dialectics of autonomy and dependence, constructiveness and destructiveness, and of the phase of identity construction where the acquisition of a stable image of the adolescent passes through comparison with other adolescents. The expression of such a condition is therefore necessarily colored by issues of group identification, leading to necessary identity wanderings—which are not necessarily a disorder.

### Neuropsychological explanations

Like patients with a DID, a number of neuropsychological dysfunctions could be detected in patients with DIC, e.g., metacognitive disorders, alexithymia, or emotional regulation difficulties (35). For instance, meta-cognitive abilities are necessary to attest to the sense of personal unity.

### Socio-cognitive explanations

In the literature, DID and DIC have been described according to a socio-cognitive model (36). In this way, highlighting a DIC or a DID in the population increases consultations and diagnoses, leading to social reinforcement in both patients and the medical community, and an inflation of prevalence. For instance, diabetes could be understood according to this socio-cognitive model: soon after the existence of diabetes was highlighted, this little-known disorder had led to an increase in consultations, which increased diagnostic expertise, leading to greater diagnostic sensitivity and more targeted diagnoses (37). This model, therefore, gives an important place to the sociocultural context in the *expression* of a condition/disorder (4). In this context, the DIC corresponds to a condition consisting of the expression of multiple social roles that have been created, legitimized, and maintained by a mechanism of social reinforcement (36).

### Emotional labeling

The labeling of “alters” (aka the identities of the system—see Table 2) could simply correspond to metaphors for different emotional states, i.e., the labeling of these states (12, 38). Especially, such labeling allows young people to legitimize a distancing between themselves and some of their emotions (39); this distancing can be beneficial to relieve (potentially without voluntary intention) responsibility about objectionable or not accepted actions or behaviors according to the values of their communities (e.g., scarifications, which can be incompatible with the values of their family, while they can be in conformity according to the values of their peer group).

Especially, as in DID, the question of depersonalization (a component of dissociation) could be found in DIC. During depersonalization, the individual experiences a loss of sense of self, of their mind and/or body. We sustain that the labeling of this emotional loss can be described as an “alter.” However, unlike the DID described in the DSM, this labeling does not only correspond to naming an emotion. On the contrary, it is part of a form of self-narrative that we will describe below.

### Narrative explanations

The possibility for a patient to maintain their narrative agency, i.e., the possibility they have of telling themself a coherent story of their own existence, could be influenced by medical accounts. For instance, the diagnostic label offers the patient a narrative coherence because it proposes an explanation of some of their demonstrations of distress (40). Losing this narrative coherence can hamper the individual story, which is the Ariadne's thread with which a person defines their self and gives meaning to their life (41). In other words, medical diagnoses result from the interaction between the narrative construction of a community (medical for the west or spiritual for other ethnic groups) and the experience of inner suffering (42). Therefore, a diagnosis such as DIC can alter the story that an individual can tell about to themself about their own life (40), in particular by reinforcing the vital sense of continuous self-narrative. Presenting a DIC supports this self-narrative by creating an identity.

### Explanations of transient illnesses

Psychiatric disorders cannot be considered as natural kinds, i.e., entities fixed in time, analyzable outside of personal experiences, and independent of the theories, models, and observation methods of clinicians and scientists (40). Conversely, psychiatric disorders are families sharing similar clinical pictures with significant heterogeneity, integrated into nosographies that may evolve over time as revisions are made (43, 44).

In order to discuss mental health, patients or associations necessarily have to use medical vocabulary, which is especially described in psychiatric nosology. Thus, they rely on the dialect used by clinicians and the vocabulary of nosographies. This dependence on representational discourse has an influence on the constitution of psychiatric disorders. The definition of these disorders partly depends on the medical discourse. In other words, the suffering of a patient should include medical representations, themselves dependent on a social and historical context. The definition of a psychiatric condition, such as DIC, is necessarily part of medical networks, communities, and institutions in mutual interactions (45, 46).

This crossed impact of medicine on representations, and of popular representations on medical descriptions corresponds to a so-called looping effect (47). As part of the looping effect, patients learn ways of talking about their suffering, adapt to them, and confirm clinicians' expectations. Then, the patients are led to a new description of their experience: they express their behavior differently; finally, when patients present with DIC, their condition is confirmed by professionals, and this validation will in turn modify the presentation of DIC in the patient, a presentation that will modify in return the representations of professionals (47, 48)—allowing the close of the looping effect (47, 49).

Talking about DIC in the international literature has an ecological responsibility, by creating a scientific niche in the ecosystem of medical knowledge on DIC. For instance, the growing increase in publications about DIC reinforces the looping effect and the institutional and community stability of this potentially non-medical condition (25).

To conclude, the increase in communications between professionals and the patient community leads to a looping effect that reinforces the presence, presentation, and stability of DIC, and thus their care and potential treatment.

In conclusion, DID as described in international classifications (ICD and DSM) seems to correspond to a diagnosis, whose psychiatric semiology is blurry, and which does not correspond to the clinical presentations encountered in adolescent psychiatry (here presented as DIC). The clinical presentations encountered in the youth clinical practice correspond to dissociative conditions of identity, resulting from a narrative interpretation of medical discourse by popular culture, associated with self-diagnosis and on the basis of probable episodes of dissociation. Such a transient disease fits in an ecological niche, which echoes the values of society, persisting under the action of a need for narrative continuity of the self. Thus, the DIC, as a conceptual entity distinct from DID, corresponds to a I presentation of the latter nourished by profane information. However, DIC is developing in patients presenting undeniable distress. In normative terms, it is important to know how to put aside any denomination (medical DID or popular DIC) to accept with legitimacy, according to a principle of validation of the moral pain experienced in psycho(patho)logy, any experience of suffering regardless of its status.

## Data availability statement

The original contributions presented in the study are included in the article/supplementary material, further inquiries can be directed to the corresponding author.

## Author contributions

CG: writing-original draft preparation and conceptualization. PE: methodology and visualization. OR: investigation, writing, methodology, and supervision. PF: supervision, methodology, validation, and editing. All authors contributed to the article and approved the submitted version.

## Conflict of interest

The authors declare that the research was conducted in the absence of any commercial or financial relationships that could be construed as a potential conflict of interest.

## Publisher's note

All claims expressed in this article are solely those of the authors and do not necessarily represent those of their affiliated organizations, or those of the publisher, the editors and the reviewers. Any product that may be evaluated in this article, or claim that may be made by its manufacturer, is not guaranteed or endorsed by the publisher.
